# In-Clinic Adolescent Peer Group Support for Engagement in Sub-Saharan Africa: A Feasibility and Acceptability Trial

**DOI:** 10.1177/2325958219835786

**Published:** 2019-03-24

**Authors:** David Barker, Anthony Enimil, Omar Galárraga, Dennis Bosomtwe, Nicholas Mensah, Sneha Thamotharan, Esther Henebeng, Larry Brown, Awewura Kwara

**Affiliations:** 1 Department of Psychiatry, Rhode Island Hospital, Providence, RI, USA; 2 Department of Psychiatry and Human Behavior, Alpert Medical School, Brown University, Providence, RI, USA; 3 Directorate of Child Health, Komfo Anokye Teaching Hospital, Kumasi, Ghana; 4 Department of Child Health, Kwame Nkrumah University of Science and Technology, Kumasi, Ghana; 5 Department of Health Services, Brown University School of Public Health, Providence, RI, USA; 6 Department of Medicine, Alpert Medical School, Brown University, Providence, RI, USA; 7 Division of Infectious Diseases and Global Medicine, University of Florida College of Medicine, Gainesville, FL, USA

**Keywords:** social support, adolescence, perceived stigma, medication concerns

## Abstract

Holding support groups with the same cohort of adolescents during clinic visits promises to increase engagement in care. Participants (N = 35 patients, aged 12-18, 50% female, from an adolescent HIV clinic in Kumasi, Ghana, were divided into 5 teams. Clinic visits were coordinated for members of each team. Team members participated in group discussions and activities while waiting to meet with their medical team. Teams met quarterly for 1 year. Participants reported benefits from talking with peers about the challenges of managing HIV. Clinic attendance improved from the preceding year (54% versus 84%). There were reductions in perceived internal stigma, perceived external stigma, worries about unintended disclosure from taking antiretroviral therapy (ART), and reduced ART concerns. The program demonstrated the feasibility, safety, and acceptability of facilitating increased interaction among adolescents living with HIV during clinic visits. Improvements in clinic attendance, perceived stigma, and concern about medications suggest that the intervention is a promising candidate for additional study.

**What Do We Already Know about This Topic?**

Adolescents living with HIV in sub-Saharan Africa have limited access to social support and struggle to attend support groups held outside of regularly scheduled clinic visits.

**How Does Your Research Contribute to the Field?**

Evaluated the feasibility, acceptability, and preliminary efficacy of an in-clinic support group for adolescents living with HIV.

**What Are Your Research’s Implications toward Theory, Practice, or Policy?**

Placing adolescents living with HIV in cohorts, synchronizing their clinic schedules, and providing them with in-clinic peer groups had minimal impact on clinic flow, was well received by patients, and showed improvements in clinic attendance, medication concerns, and perceived stigma.

## Introduction

Roughly 2.1 million adolescents are now living with the HIV,^1^ most of whom acquired the virus through vertical transmission.^2^ Among adolescents living with HIV (ALH; aged 15-24), approximately 80% live in sub-Saharan Africa, including Ghana which has approximately 24 806 ALH.^2–4^ For ALH, adolescence is a period of particular risk due to dynamic changes in physical, emotional, cognitive, and social development.^5–7^ However, these dynamics also make adolescence a critical period for intervention during which youth build disease management patterns that carry into adulthood.^6,7^

Retention in care and adherence to antiretroviral therapy (ART) among ALH are well-documented problems that adversely affect the health of ALH (eg, immunologic failure, disease progression) and increase the risk of transmission.^8–10^ Barriers to both retention in care and treatment adherence include HIV-related stigma, misinformation, limited social networks, and logistical challenges such as transportation and school schedules.^9^ Diffusion of innovations theory suggests that youth are more likely to adopt healthy behaviors when they see peers in leadership roles modeling the behavior, when they understand the benefits of the behavior relative to alternatives, and when they are able to observe others who are similar to them adopt the behavior.^11^ Unfortunately, with HIV care, stigma narrows the peer network with which ALH can discuss their illness. Interventions targeting peer support in HIV-infected populations can be instrumental in reducing stigma, increasing HIV-related knowledge, increasing motivation to engage in care, and improving outcomes.^12,13^ However, groups designed to build peer support often meet outside of regularly scheduled medical appointments, which reduces attendance, especially among those who are most in need of additional support, and adds cost to sustaining group interventions over time.

In sub-Saharan Africa, time spent waiting for a medical appointment is an underutilized resource that with modest structure and planning could provide ALH an opportunity to connect with affected peers. Regularly scheduled clinic visits often last 3 to 4 hours with much of that time spent in the waiting room. In general, youth do not engage with peers during clinic visits and thus do not form connections with their HIV-infected peers. On the few occasions that they do develop informal peer support during clinic visits, these interactions are unsupervised and have the potential of disseminating unhealthy information or modeling unhealthy behaviors (eg, avoiding medication to fit in with healthy peers). Providing a formal, supervised structure of cohorts or “teams” of youth who are consistently scheduled on the same day for follow-up appointments provides the opportunity for ALH to meet with affected peers while reducing the burden of additional travel required to attend support groups scheduled outside of normal clinic attendance. The need for interventions designed for ALH in low- and middle-income countries is high, with a recent systematic review only identifying 5 trials that included adolescents and adults and 2 that focused only on treatment adherence among adolescents.^14^ The numbers are similar for studies focusing on policies to enhance retention in care, with 11 including adolescents and adults and 1 focusing only on adolescents.^15^

The objective of this study was to evaluate the feasibility, acceptability, and preliminary efficacy of forming clinic-based peer groups in an adolescent HIV clinic in Kumasi, Ghana. The ALH were divided into mixed age and gender groups of 7 members (most support groups for adolescents range in size from 5 to 9 participants),^16^ clinic scheduling was synced for each group, and they participated in formal group-based activities while waiting for their turn to meet with the medical team. Outcomes of interest were as follows: (1) impact on clinic flow, (2) focus group reports of acceptability, and (3) impact on health outcomes, ART knowledge and attitudes, social support, perceived stigma, medication concerns, and concerns about disclosure.

## Methods

### Participants

Participants were 35 ALH from an adolescent HIV clinic at Komfo Anokye Teaching Hospital (KATH) in Kumasi, Ghana. All adolescents aged 12 to 19 years receiving treatment at the clinic who had perinatally acquired HIV were eligible to participate. Half (53%) of the participants were female; their mean age was 14.77 years old (standard deviation [SD] = 1.66); 9% were classified as World Health Organization (WHO) stage 1, 34% as stage 2, 37% as stage 3, and 20% stage 4. Ninety-one percent were on first-line treatment (abacavir [ABC] or [zidovudine, ZDV] + lamivudine (3TC) + efavirenz [EFV] or ABC [or ZDV] + 3TC + nevirapine [NVP]), and 54% were virally suppressed (viral load [VL] ≤40 copies/mL) at enrollment. The KATH adolescent clinic began in 2011 and was providing care to 180 adolescents at the time of the study. The clinic uses the same facilities as the pediatric and adult clinic but is held on separate dates and times from the other clinics. Most patients were perinatally infected and referred to the adolescent clinic from the pediatric clinic. Patients who age out of the adolescent clinic are seen in the adult clinic. Adolescent patients are scheduled for quarterly appointments when healthy and receive counseling around medication adherence when needed. There is also a monthly social support group that has 10 to 15 youths who attend regularly. Clinic is held in the mornings, and patients are seen on a first come, first served schedule with wait times often exceeding 3 hours. Our previous work has shown that a significant portion of youths seen in the clinic are not virally suppressed (62% unsuppressed), that they have prominent gaps in their HIV knowledge, and that they report limited treatment-related social support, with most reporting only 1 or 2 people who know about their diagnosis.^17^

### Study Design and Data Collection

The study protocols were approved by all institutional review boards of KATH, Kumasi, Ghana (CHRPE/AP/314/14) and Rhode Island Hospital, Providence, Rhode Island (FWA00001230). Medical staff fluent in both Twi and English recruited adolescents during routine clinic visits. Parents and adolescents consented and assented, respectively, using written consent and assent. Adolescents were divided into 5 groups by the clinical team. Clinic staff determined team membership after considering the patient’s next appointment date, age, gender, and disease severity. The goal was to include adolescents of different ages, genders, and disease severity in each team to help provide each team with a diversity of perspectives and lived experiences as well as provide mentoring and leadership opportunities for older adolescents consistent with diffusion of innovations theory. The team also aimed to balance age, gender, and disease severity across teams, while considering the need to minimize disruption to participants’ typical clinic scheduling. Quarterly clinic visits were synced for members of each team. Having mixed age and gendered groups greatly facilitated the synching of schedules without undue acceleration or delay in participants’ regularly scheduled visits.

Participants completed paper–pencil questionnaires prior to the start of each of 4 quarterly team meetings, with longer assessment batteries given at the first and fourth meeting. After completing questionnaires at each clinic visit, participants were compensated US$15 for their effort and travel. Administration was in written English in a private environment. For those who had difficulty reading or who were more comfortable with the local language (Twi), study staff orally administered the questionnaires in Twi. Translation from English to Twi was performed by consensus among local study staff and the lead local investigator of the study (A.E.). Results report on data collected from questionnaires completed before the first and fourth team meeting. Questionnaires were selected after a comprehensive review of the literature and focused on questionnaires that were closely aligned to the outcome of interest, relatively brief to enable routine assessment during clinic visits, easy to read (ie, <seventh-grade reading level), and ideally used in similar populations.

### Intervention

Our intent was to evaluate the feasibility and acceptability of running a support group during regularly scheduled clinics. The decision to insert the group within the clinic was to help overcome barriers to access (eg, transportation costs, perceived stigma) that limited the use of out-of-clinic support groups. Our interest was less about the content of the support group meetings and more about the structure of how standard support group materials could be delivered during clinic hours. We thus used a resource developed by multiple international agencies to facilitate dissemination of best practices in conducting adolescent peer support groups.^18^ This resource was used in the training of facilitators and in guiding the content and structure of group activities and discussions.

Using mixed age and gender groups was informed by the diffusion of information theory, which describes how peer groups influence group members.^11^ The theory suggests that building supportive norms around positive health behaviors, working with a peer leader to model healthy behavior, and build group cohesion so that members identify with the group will encourage the adoption of positive health behaviors such as medication adherence. Consistent with this theory, patients were oriented to the purpose of the team, helped define norms for appropriate team behavior, selected a team name to provide a sense of identity, and elected a team leader. Team leaders were provided with prepaid phone credits to check-in with consenting team members between clinic visits. Team leaders were given additional training on the importance of adherence to antiviral medication, the importance of avoiding unintended disclosure during telephone communication, and on how to appropriately motivate team members.

At each clinic visit, the teams met together with 2 facilitators. Facilitators were 4 bachelor-level health-care professionals who were already working in the clinic as counselors or research assistants. Facilitators were trained according to current recommendations to provide a positive, supportive, and interactive group environment that facilitated group discussion. Facilitators then used modules from the clinical resource mentioned earlier to lead the group discussions.^18^ Modules were selected to focus on topics relating to engagement in care and treatment adherence. All teams received modules on treatment adherence and understanding HIV. Team facilitators selected from additional modules based on the needs of the team (eg, making decisions and planning for the future, handling stigma and discrimination, communication and problem-solving, and disclosure/developing trust in relationships).

Clinic flow was altered so that the first portion of the clinic was dedicated to urgent and other nonregular appointments, during which time teams would meet together to participate in the team building activity. The second portion of the clinic was dedicated to the participants, and team activities proceeded as participants rotated in and out of the activities to meet with medical providers. Participants who arrived early would wait for a majority of the group to arrive at which point the team building activity would begin. On average, groups met together for 2 hours per visit for 4 visits.

### Qualitative Assessment and Analysis

All teams participated in focus groups at the end of the last clinic visit to provide feedback on their experience. Participants were not required to answer any questions they were uncomfortable with. In addition, to ensure patient comfort and openness, trained research assistants administered the groups in the native language. To avoid biases, these research assistants were previously unknown to the participants. Five focus groups were conducted in a private location at the HIV clinic, with groups ranging from 5 to 7 adolescents. The group discussions were guided using a semistructured interview guide that focused on 4 topics: barriers to clinic attendance, barriers to treatment adherence (eg, What are the issues at home, school, or work that make it difficult for you to attend clinic [take your pills]?), adolescents’ perspective of how the in-clinic peer groups helped them stay healthy (eg, How did the group help you to stay healthy?), and ways to improve the program (eg, How can we make this program better?). These discussions lasted approximately an hour and were audio-recorded. The lead investigator (A.E.) and clinic director listened to the audio recordings and provided written and translated summaries for review by the entire research team. Participants who were not able to attend focus groups participated in an individual interview.

### Quantitative Measures and Analysis

#### Clinical/Health Outcomes

Clinical/health outcomes included clinic attendance, WHO clinical staging, complete blood count, CD4 count, and VL. Chart review was used to determine attendance for the year prior to the study and to provide clinical histories for each participant. Blood samples were collected at the first and last clinic visit. The ART resistance was performed using blood samples collected at the last visit for participants who were not virally suppressed at that assessment (n = 12). All blood work was performed by Rayben Diagnostics Ltd Kumasi, Ghana, and HIV resistance testing at Labor Dr Wisplinghoff diagnostic services in Köln, Germany (http://www.wisplinghoff.de).

#### Antiretroviral Therapy Knowledge and Attitudes

The 16-item validated questionnaire assessed ART knowledge, attitudes, and beliefs: 7 items addressing knowledge of ART benefits or side effects (eg “HIV can be controlled by ART”; Cronbach α = .29), 3 items addressing the importance of ART adherence (eg, “Taking ART on schedule prevents you from being sick”; α = .52), 3 items addressing worries about others finding out they are on ART (eg, “Are you worried about friends finding out you are on ART?”; α = .51), and 3 items addressing worries about ART effectiveness (eg, “Are you worried about ART side effects?”; α = .66).^19^

#### Medical Outcomes Study Social Support Survey

The survey assessed individuals’ perceptions about available emotional/informational support (8 items), tangible support (4 items), affectionate support (4 items), and positive social interaction (4 items).^20^ The Medical Outcomes Study was specifically developed for patients with chronic conditions to evaluate functional social support. Cronbach α was .83.

#### Perceived HIV-Related Stigma

Participants reported on both internal and perceived external HIV stigma.^21^ The internal stigma scale measured the adolescent’s actual emotions or experiences related to having HIV (eg, “I feel I am not as good a person as others because I have HIV”). In contrast, the externalized stigma scale required participants to think of the feelings or behaviors of others (eg, “People I know would treat someone with HIV as an outcast”). Higher scores indicate experiencing greater amounts of stigma. Cronbach α for the internal stigma scale and externalized stigma scale were .70 and .87, respectively.

#### Safety

Participants completed the16-item Multidimensional Peer-Victimization Scale,^22^ a measure of peer victimization, at the beginning of each team meeting. The measure is a checklist that assesses physical victimization, verbal victimization, and social manipulation. The measure is not HIV-specific and was intended to be sensitive to any teasing, bullying, or other unwanted behavior that was being experienced by participants. Participants were also privately queried following each team meeting if they experienced any teasing, bullying, or other unwanted behavior from group members. Any endorsement of unwanted behavior was followed up by study personnel following a prespecified procedure and the clinic director (A.E.) was informed so that appropriate actions or referrals could be taken if needed.

#### Analysis Plan

Missing data, which were 11% for self-report measures and 17% for biologic outcomes, were addressed using multiple imputations using chained equations.^23^ Results from each of 50 imputed data sets were analyzed using generalized estimating equations, with an exchangeable working correlation structure, to account for nesting of assessments within participants. Team membership was added as a categorical covariate to address nesting of participants within adherence teams. Effect sizes were estimated using odds ratios and rate ratios for dichotomous and count outcomes and standardized difference scores for continuous outcomes. Addressing missing data is challenging in small samples, and while the performance of missing data models may suffer in small samples, our selected approach has been shown to perform comparably to alternative approaches with slightly higher power and robustness when assumptions are not met.^24^ The percentage of model uncertainty accounted for by the missing data models ranged from 4% to 15% for self-report measures and from 10% and 27% for biological outcomes, with the 27% being for detectable viral load. Analyses utilized the following R packages: *mice* v2.25 and *gee* v4.13.

## Results

### Feasibility and Acceptability

The clinical team informally reported that altering the clinical scheduling and facilitating the in-clinic peer groups had little impact on overall clinic flow. Since patients are served on a first come, first served basis, the medical providers were able to address the needs of patients who were not participating in the groups during the first portion of the clinic, while teams participated in team building activities. Participants rotated in and out of the group during the second portion of the clinic to meet with medical providers. Attendance by ALH in the clinic was higher on days when one of the 5 peer groups met (11.4 patients) versus on typical clinic days (6.7 patients), likely due to the increased attendance among group members. The increased attendance was viewed positively by the clinical team and did not impact their ability to finish the clinic on time.

Themes from the qualitative analysis are reported in Table 1. Generally, participants found the groups to be supportive and enjoyable with no reports of disrespectful or coercive behaviors among group members. They enjoyed the ability to connect and learn from each other not having to worry about HIV-related stigma. Specifically, they reported that the facilitators were supportive (eg, “Group leaders advocated for us”), groups fostered communication among group members (eg, “Before the initiation of the group, there was no communication when we visit the clinic”), provided valuable education (eg, “I was at first defaulting medication but have learn through the group that the VL would increase if I do not adhere”), provided opportunities to build friendships, were supportive and accepting, and helped motivate them to stay healthy (eg, “Through our discussion we encourage group member to strive hard to achieve our goals in life. Such message keeps us healthy” and “Commending those who adhere to the pill, motivate me to take mine”). When asked for suggestions about improving the program, they suggested adding tangible incentives or competition with other groups as a way to provide additional motivation. They also indicated a need for frequent reminders to take their medication and advice on how to stay healthy.

**Table 1.** Qualitative Themes.

Categories	Themes	Examples
Clinic attendance	Conflicts with other priorities	School examination
	Transportation issues	Traffic, 1- to 2-hour commute, no car
	Scheduling	Long waits, first come first served makes for unpredictable wait time
	Stigma	Sometimes we are punished for being absent because we can’t say our conditions made us go for drugs
Medication/adherence	Financial constraints	No money for transportation, or labs
	Lack of awareness/knowledge	“I didn’t know the reason for taking the medication,” “At times I become worried and confused why I got infected in the first place because I am not sexually active”
	Concerns about disclosure	Prefer taking meds in the evening when no one is around, “school mates ask me lots of questions when I take the drug at school,” “sharing room with sibling who doesn’t understand what I take medications often,” and “we should be given container to keep the pill that would be taken outside the house”
	Availability	Drug shortages, “drugs are changed frequently,” have to wait long to be served, should be made once a day
	Self-sufficiency/dependency	Determined to be responsible for own health, “my life depends on it”
	Taste	Bitterness
	Side effects	Difficulty in concentrating in class, feel drowsy, paints in throat
	Conflicts with other priorities	duties at the shop, school sessions, school exams, running errands
	Food	Scarcity, eat heavily before taking the pill
	Forgetfulness	Forget to take when traveling alone, if late for school, set alarm clock as reminder
In-clinic peer groups	Supportive facilitators	Taught how to take pills, provided encouragement, given reminders about next clinic visit
	Fosters communication	Helps with public speaking, encouraged to stay healthy, able to talk about HIV without stigma
	Educational	Learned how to take medication, discuss health issues, share best way of taking drugs to be healthy Learned how taking medications can improve health
	Built Friendships	“Didn’t know anyone at clinic but now we have lots of friends” “When I see my friends doing well with the medication, it motivates me”
	Love and acceptance	Felt loved, no stigma in group, group members love and motivate each other, respectful, hold each other accountable
	Motivation	“When I see my friends doing well with the medication, it motivates me to do so,” “Commending those who adhere to the pill, motivate me to take mine”
Ways to improve groups	Incentives	“If group is given a prize it will motivate us all to come for clinic and also to take our pills.”
	Frequency	“Providing frequent advice helps us to take our pills,” “Enquire frequently about taking the pills will motivate us”
	Competition	“We should organize quizzes with other groups,” “Competition will improve adherence”

### Safety

Safety was assessed through individual questions following each team meeting and through a formal assessment of aggressive behavior. There were no reports of physical victimization, verbal victimization, or social manipulation by team members either during group meetings or in the period between meetings.

### Preliminary Efficacy

**Figure 1.**
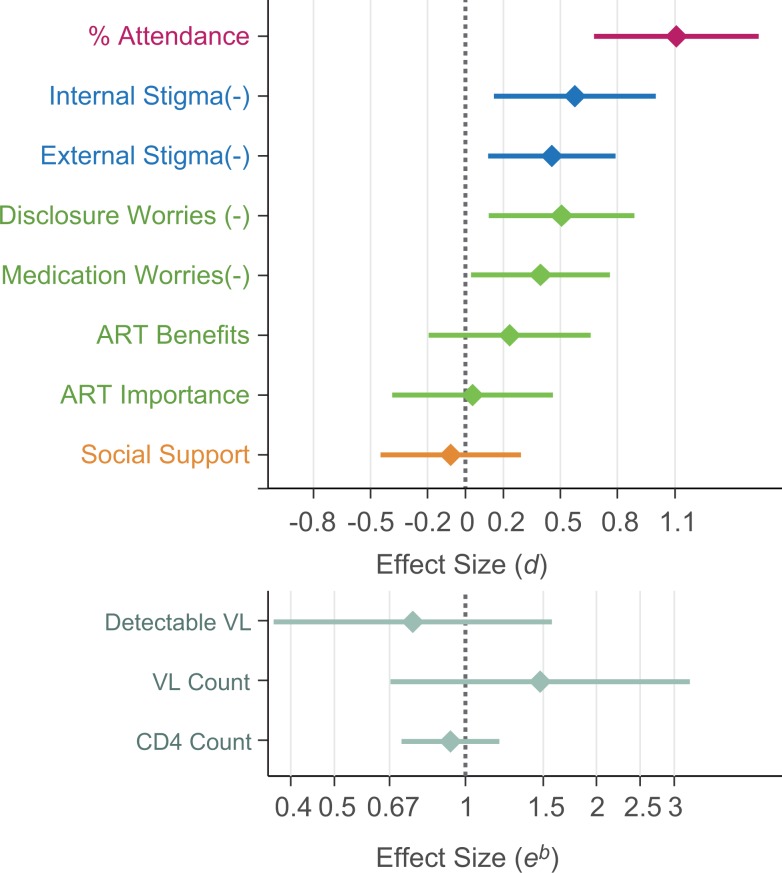
Adjusted effect size estimates. Effect sizes and 95% confidence intervals for change from baseline to the 9-month assessment. *Note:* Attendance during the program was compared to attendance during the year preceding baseline. (−) measures were reverse coded so that positive scores indicate healthier response. ART indicates antiretroviral therapy; VL, viral load; *d*, standardized difference from baseline; *e^b^*, odds ratio for binary and rate ratio for count outcomes.

Unadjusted and nonimputed descriptive statistics are presented by assessment in Table 2. Imputed and adjusted effect sizes and confidence intervals are presented in Figure 1. Results showed a large effect size for improved attendance (*d* = 1.13 [95% confidence interval = 0.69 to 1.13], *P* < .01), medium effect sizes for reduced perceived internal stigma (*d =* −0.56 [−0.98 to −0.14], *P* < .01), reduced perceived external stigma (*d =* −0.44 [−0.78 to −0.11], *P* < .01), reduced worry about unintended disclosure from taking medications (*d =* −0.51 [−0.89 to 0.12], *P* = .01), and reduced worry about ART medications (*d =* −0.40 [−0.76 to −0.03], *P* = .03), and small effect sizes for improved belief in ART benefits (*d =* 0.23 [−0.18 to 0.65], *P* = .27). There were no changes, however, in the importance for ART adherence (*d =* 0.04 [−0.38 to 0.46], *P* = .85), number of virally suppressed participants (odds ratio = 0.76 [0.36 to 1.59], *P* = .46), VL (rate ratio = 1.49 [0.67 to 3.30], *P* = .33), CD4 count (rate ratio = 0.94 [0.75 to 1.17], *P* = .56), or total social support (*d =* −0.06 [−0.41 to 0.28], *P* = .72)

**Table 2.** Unadjusted Descriptive Statistics.

Mean (SD) or % (n)	Baseline	9 Months
	n = 35	n = 31^a^
Clinic attendance (% of quarterly visits during previous year)	53.60 (27.20)	84.30 (25.10)
Perceived stigma		
Internalized stigma	2.06 (0.52)	1.76 (0.32)
Externalized stigma	3.76 (0.70)	3.45 (0.41)
ART		
Worries about unintended disclosure	2.34 (0.91)	1.86 (0.43)
ART adherence concerns	2.15 (1.04)	1.71 (0.47)
Benefits to taking medication	0.76 (0.17)	0.80 (0.16)
Importance of taking medication	0.89 (0.23)	0.91 (0.15)
Social Support		
MOS total support	61.7 (13.67)	62.9 (15.52)
Medical		
Detectable viral load	46% (16)	41% (12)
Log_10_ viral load	2.51 (1.14)	2.52 (1.20)
CD4 count	552 (344)	519 (362)

Abbreviations: ART, antiretroviral therapy; MOS, Medical Outcomes Study; SD, standard deviation.

^a^Medical Outcomes *n* = 29.

### Antiretroviral Therapy Resistance Test Results

The resistance profiles of the 12 participants who showed detectable VL at the 9-month assessment are as follows: 3 had resistance to EFV (K103 N or K103 N & P225 H mutations), 3 had resistance to both 3TC (M184 V) and EFV (K103 N & P225 H), 1 had resistance to 3TC alone (M184 V mutation), and 1 had no resistance mutations. Polymerase chain reaction (PCR) amplification failed in the other 4 patients. In summary, 7 participants in whom PCR was successful showed some resistance mutation that likely impaired ART efficacy.

## Discussion

Among ALH in sub-Saharan Africa, barriers to both retention in care and treatment adherence include HIV-related stigma, misinformation, limited social networks, and logistical challenges such as transportation and school schedules.^9^ Results from the qualitative interviews suggest that our sample reported similar barriers to retention in care and treatment adherence. This study evaluated the feasibility, acceptability, and initial efficacy of an in-clinic peer group that met quarterly for 9 months. Results indicated that changing clinic scheduling so that ALH were grouped into groups that shared the same clinic schedule was feasible and had minimal influence on clinic flow. Requirements to run the in-clinic peer groups were modest and included space for the group to meet, 2 entry-level health-care professionals to be present during the clinic, and minimal supplies (eg, pictures, notebooks, flip-chart) for running group activities. The ALH reported enjoying discussing their illness with peers in an accepting, stigma-free environment.

In terms of quantitative outcomes, participants reported marked improvements in perceived internalized and externalized stigma, reduced concerns about others finding out they were on ART, reduced concerns about their medications, and improved clinic attendance. The magnitude of the effect sizes suggest that the intervention holds promise in reducing perceived stigma and concerns about medications. These quantitative results align with the qualitative themes identified from the focus groups in that participants reported enjoying supportive discussions about HIV free from stigma and that they learned more about their medications and were motivated to adhere by listening to the perspectives of their peers. Reducing perceived stigma has benefits outside of the well-being of ACH. With lower perceived stigma, ACH may be more likely to inform others about their illness, which has been shown to lower community-level stigma.^25,26^ Contrary to expectations, there were no changes in CD4 count or log_10_ VL. The small effect sizes from these null findings suggest that the intervention may not have been sufficiently powerful to change treatment-related behaviors. Group cohesion is an important mechanism linked to positive outcomes for therapeutic and support groups. It is possible that the ability of the intervention to generate cohesion was adversely affected by the low number of team meetings (3 meetings prior to the final assessment that occurred prior to the fourth team meeting) or by the 3-month interval between visits. Meeting quarterly may not be sufficiently frequent to build levels of group cohesion necessary to motivate improvements in treatment adherence or to sustain potentially supportive team relationships outside of clinic. It is also possible that ineffective therapies due to preexisting drug resistance at enrollment may have obscured changes in treatment adherence, which was not directly measured in this study.

There are a number of possible ways to boost the potency of the in-clinic peer groups. First, the quarterly group meetings in the community could supplement the quarterly visits. While this approach increases the frequency of meetings, it may be problematic for adolescents to travel to attend the meeting, particularly for those who are in boarding school, which is a common educational arrangement for secondary education in Ghana. Second, clinics could use social media platforms to encourage interactions outside of the clinic. Adolescent clinics in Ghana already use platforms such as WhatsApp to connect with ALH. However, many ALH do not use these social media platforms, particularly those who are in most need of support. Third, clinicians could consider geographical location when forming teams, better enabling adolescents to meet on their own outside of clinic. Anecdotal evidence suggests some ALH have benefited by having a “big brother” or “big sister” at their school to help them manage their medications. On the other hand, other ALH do not want anyone in their school or neighborhood to know about their diagnosis. It is important to consider privacy concerns before using geography when forming the groups. Finally, additional strategies could be used to facilitate and enhance group cohesion during the group visits. Providing groups with a shared goal (eg, lower group viral load) or sense of purpose (eg, advocacy work or community engagement) that extends outside of the clinic may help encourage contact outside clinic, increase motivation to attend clinic, and work toward the common goal. Group-based economic incentives or between-group competitions could be used to help build a shared goal or sense of purpose.

There are a few limitations to consider when interpreting the results from this study. First, participants received reimbursement for completing study questionnaires during clinic visits, which likely served as an individual economic incentive contingent on attendance. The current design could not separate the influence of the reimbursement from that of the in-clinic peer groups. Therefore, the effect on clinic attendance should be interpreted as a result of both an individual economic incentive and participation in the in-clinic peer groups. Second, there was no comparison group, limiting conclusions about the efficacy of the in-clinic peer groups. Third, there was not a direct measure of treatment adherence, which greatly limited our ability to disentangle self-care behavior from the efficacy of participants’ ART regimen. Fourth, there was no baseline resistance testing of the adolescents who had detectable VL at enrollment to enable a change to second-line effective regimen. Thus, improvement in treatment adherence due to the intervention may not have reflected in VL suppression. Finally, the mixed age and gender groups limit the scope of topics available for discussion. For example, it would be difficult to discuss sexual risk behavior or romantic relationships in such groups. Future work may consider separating groups by age and/or gender. Although these limitations suggest caution in interpreting the efficacy of providing in-clinic peer groups, results are promising, particularly in increasing clinic attendance and reducing perceived HIV-related stigma.

## Conclusion

This study demonstrated the feasibility of implementing an in-clinic peer group in a resource-limited setting that the program was enjoyed by ALH and that there were no observed or reported instances of unwanted interactions among groups that included a mixture of gender, age, and illness severity. The feasibility, acceptability, and safety together with improvements in clinic attendance, perceived stigma, and concern about medications suggest that in-clinic peer groups may be a cost-effective approach to improving connections among ALH. Providing opportunities to connect with peers who share their experience may be particularly important in areas where both HIV stigma and economic hardship reduce the availability of caring individuals with whom ALH can share their experiences and frustrations. More work is required to further validate in-clinic peer groups and better understand how they might influence engagement in care and treatment adherence.
